# Supplementary figures and images for: A Commander-independent function of COMMD3 in endosomal trafficking
Source: eLife. 2025 Aug 21;14:RP105264. doi: 10.7554/eLife.105264 (PMC12370252; doi:10.7554/eLife.105264)

# Fig. 2a

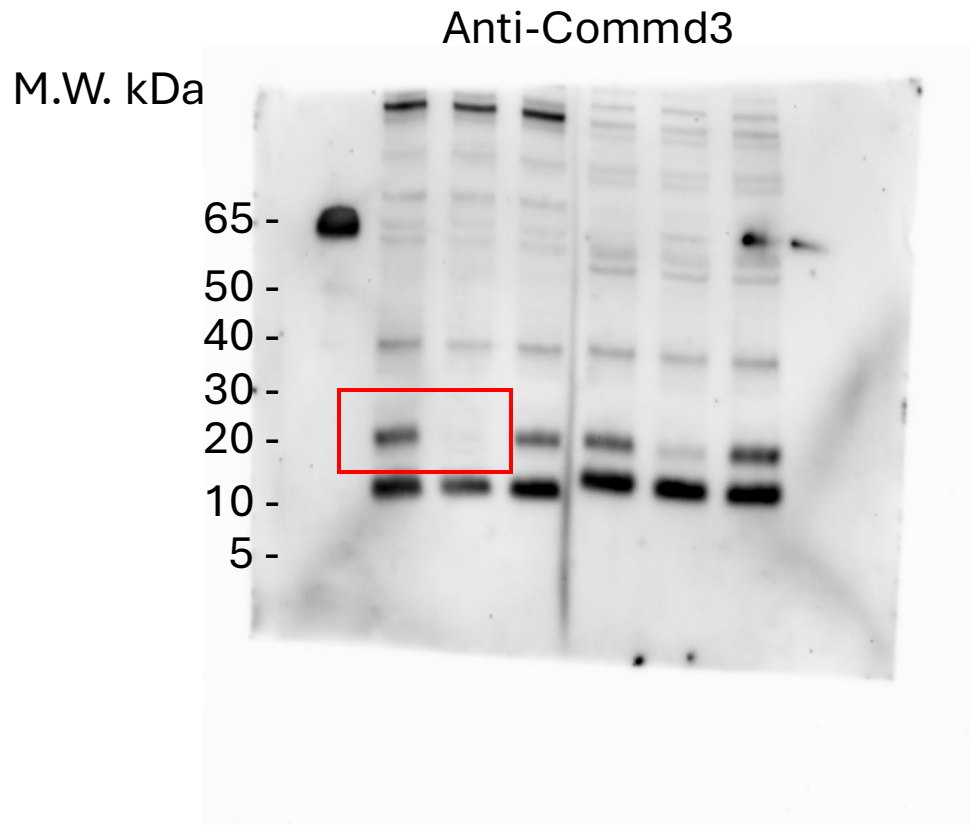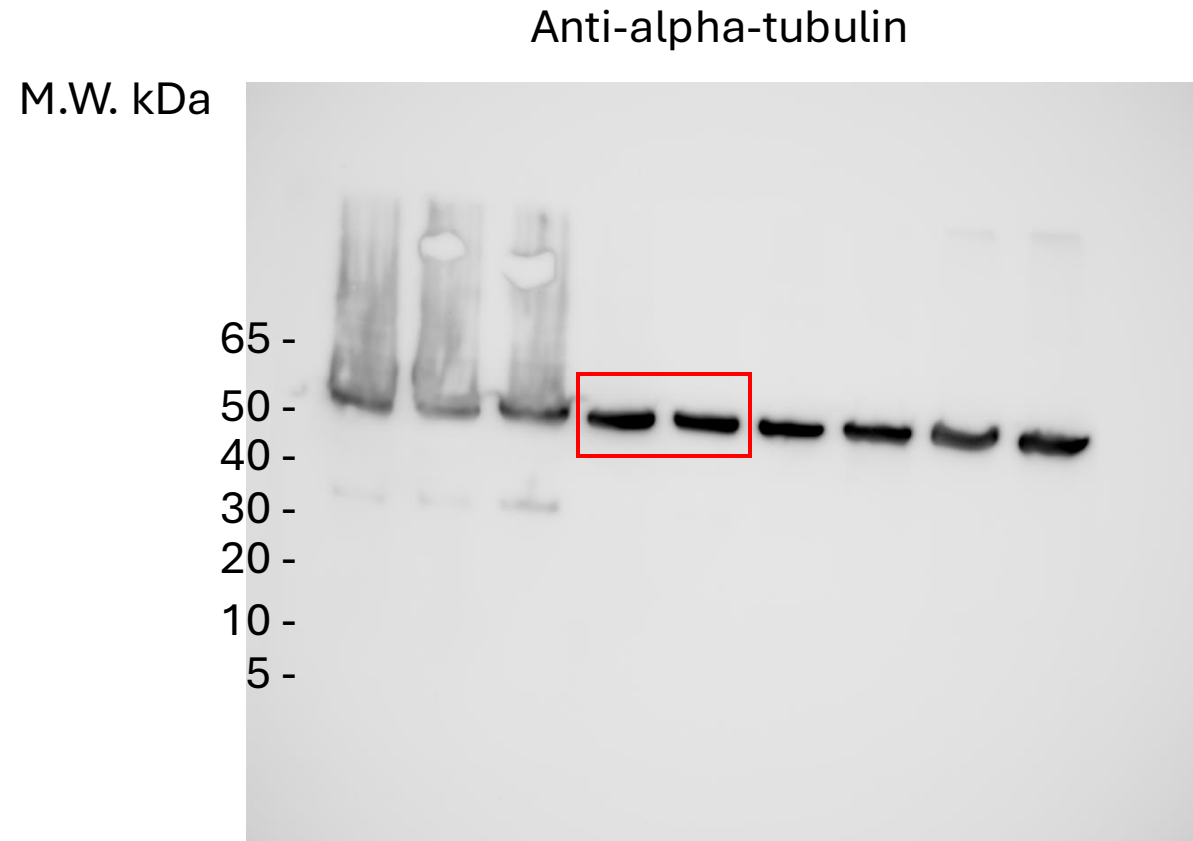

Supplement: Figure 2—source data 1. [file elife-105264-fig2-data1.zip › Figure 2-Source data1.pdf]

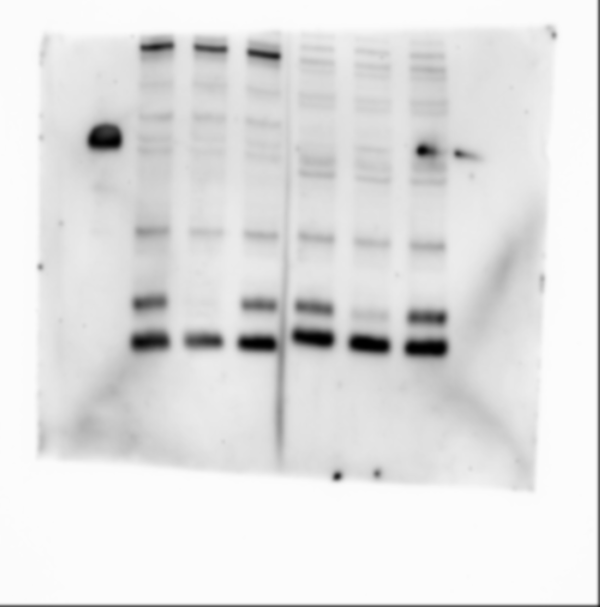

Supplement: Figure 2—source data 2. [file elife-105264-fig2-data2.zip › Fig2a anti-Commd3.tif]

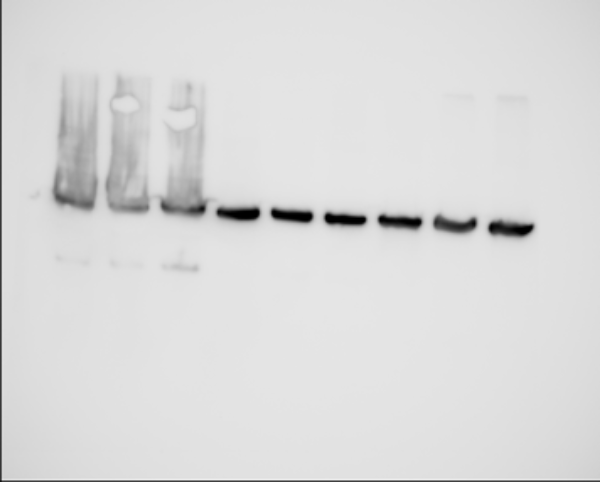

Supplement: Figure 2—source data 2. [file elife-105264-fig2-data2.zip › Fig2a anti-Tubulin.tif]

# Fig. 5a

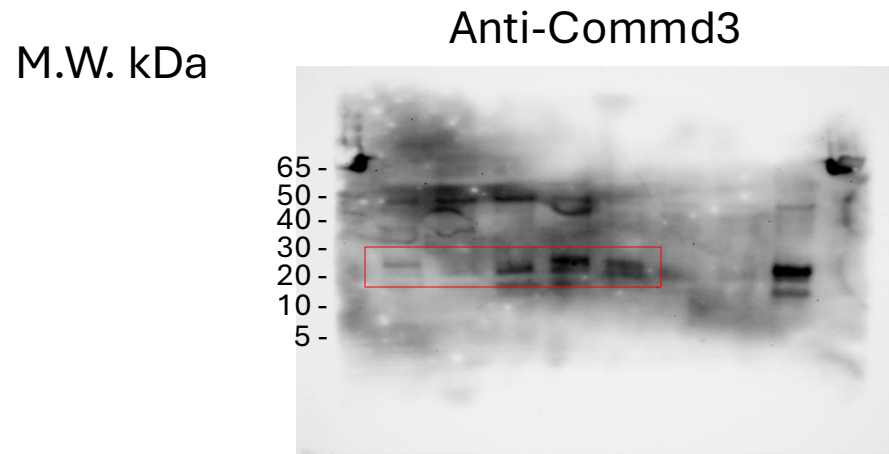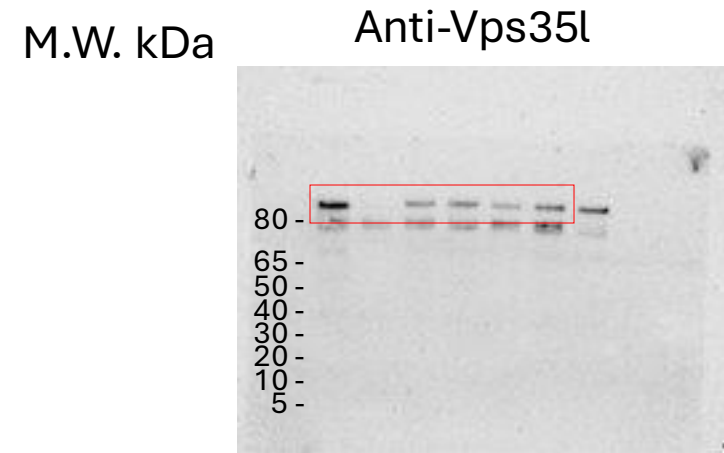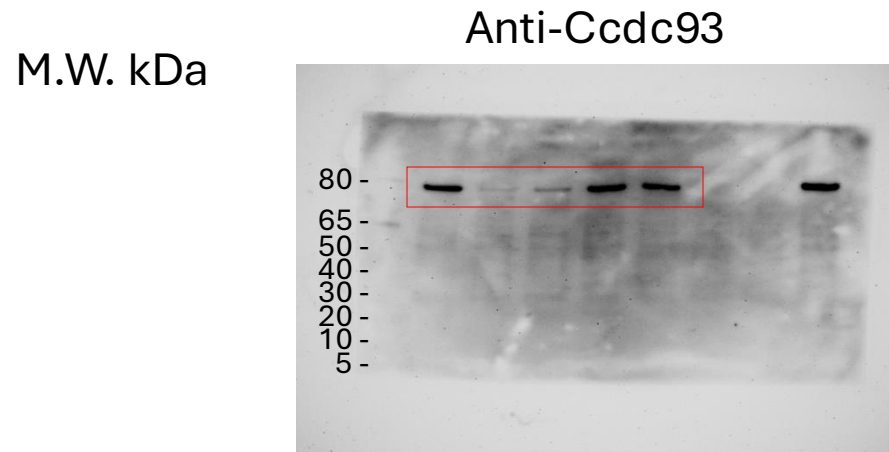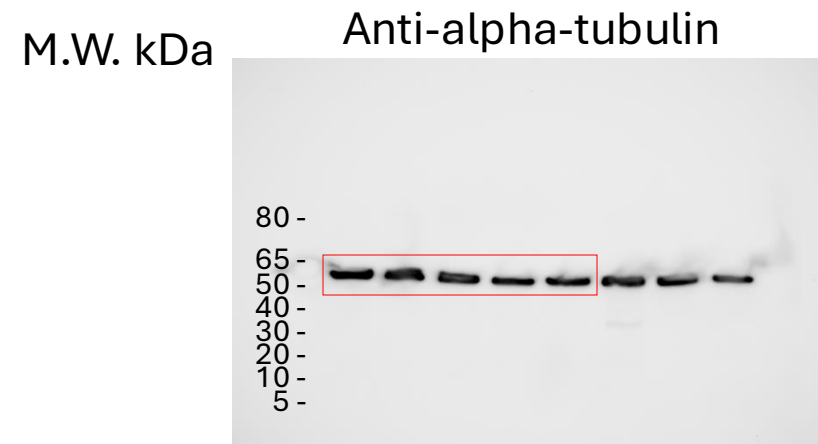

Supplement: Figure 5—source data 1. [file elife-105264-fig5-data1.zip › Figure 5-Source data 1.pdf]

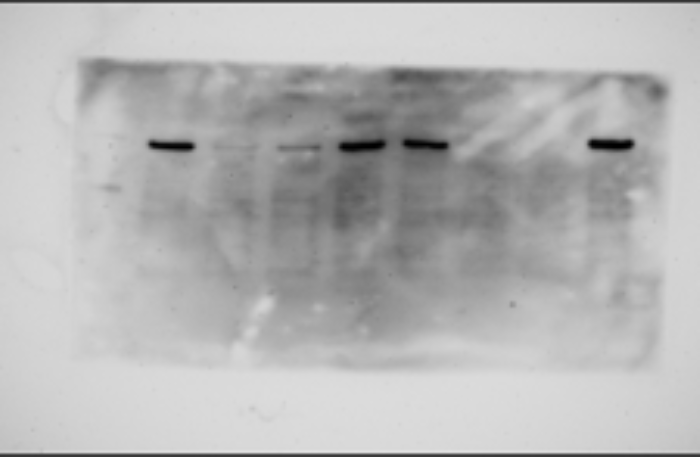

Supplement: Figure 5—source data 2. [file elife-105264-fig5-data2.zip › Figure 5a anti-Ccdc93.tif]

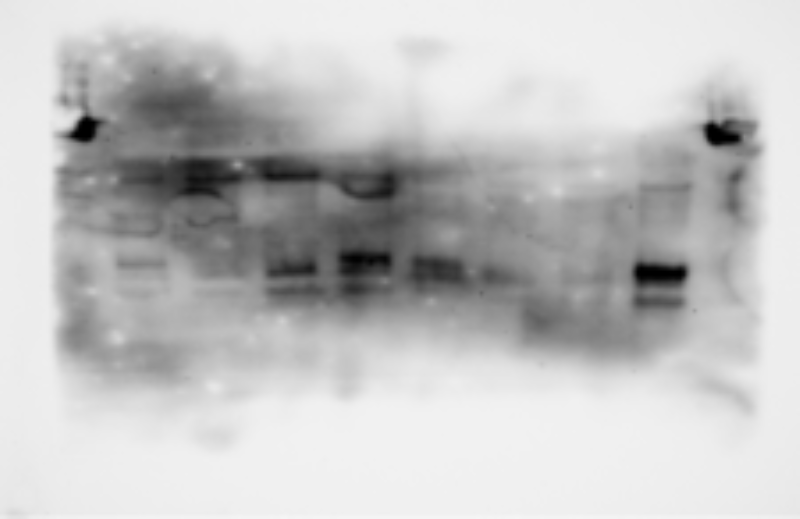

Supplement: Figure 5—source data 2. [file elife-105264-fig5-data2.zip › Figure 5a anti-Commd3.tif]

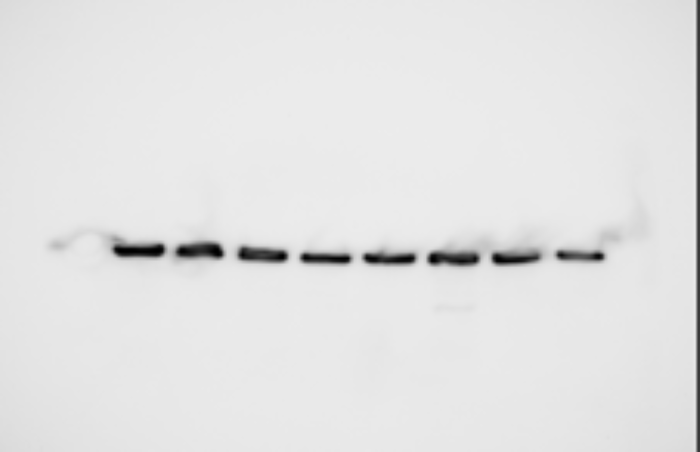

Supplement: Figure 5—source data 2. [file elife-105264-fig5-data2.zip › Figure 5a anti-Tubulin.tif]

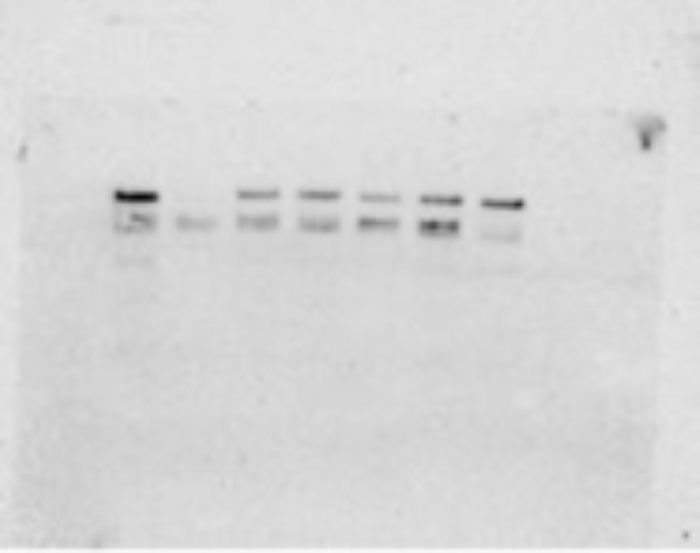

Supplement: Figure 5—source data 2. [file elife-105264-fig5-data2.zip › Figure 5a anti-Vps35l.tif]

# Fig. 6g

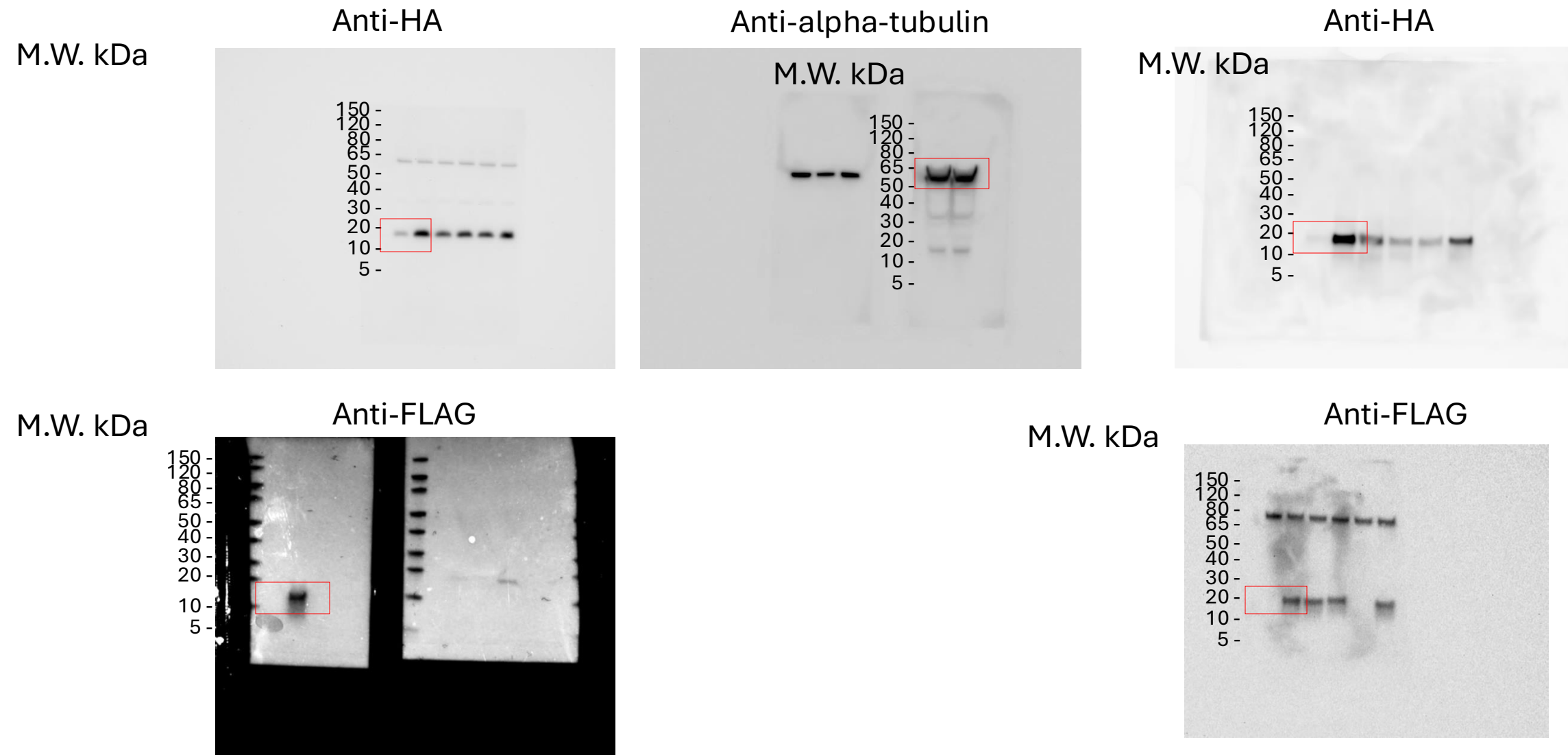

Supplement: Figure 6—source data 3. [file elife-105264-fig6-data3.zip › Figure 6-Source data 1.pdf]

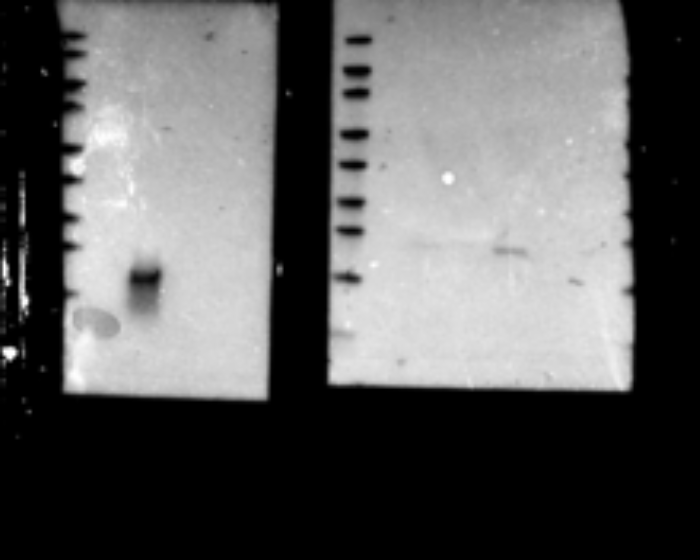

Supplement: Figure 6—source data 4. [file elife-105264-fig6-data4.zip › Figure 6g anti-FLAG.tif]

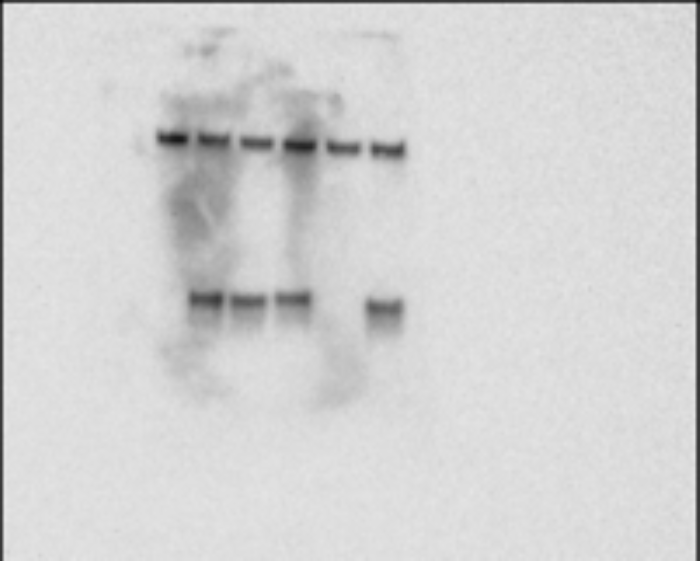

Supplement: Figure 6—source data 4. [file elife-105264-fig6-data4.zip › Figure 6g anti-FLAG2.tif]

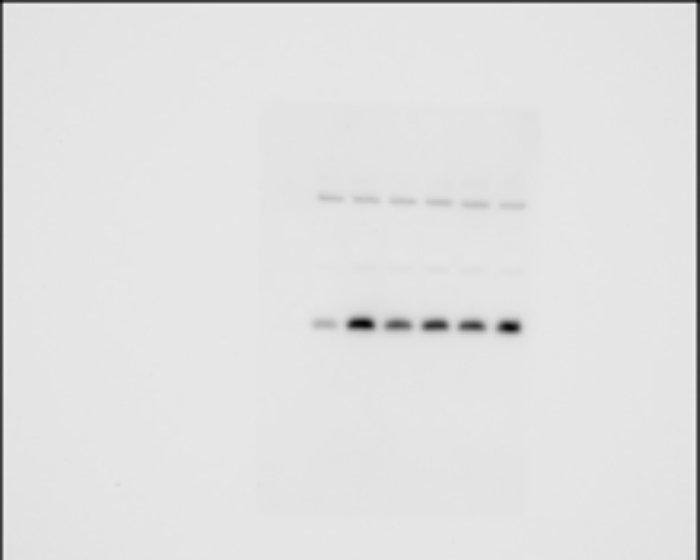

Supplement: Figure 6—source data 4. [file elife-105264-fig6-data4.zip › Figure 6g anti-HA.tif]

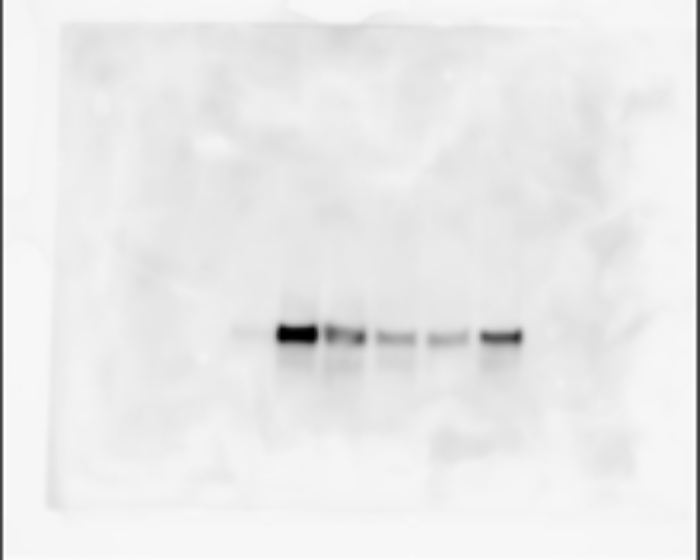

Supplement: Figure 6—source data 4. [file elife-105264-fig6-data4.zip › Figure 6g anti-HA2.tif]

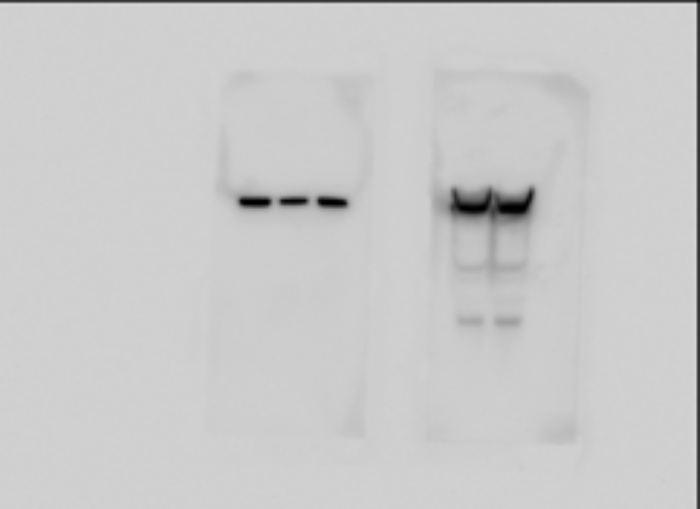

Supplement: Figure 6—source data 4. [file elife-105264-fig6-data4.zip › Figure 6g anti-Tubulin.tif]

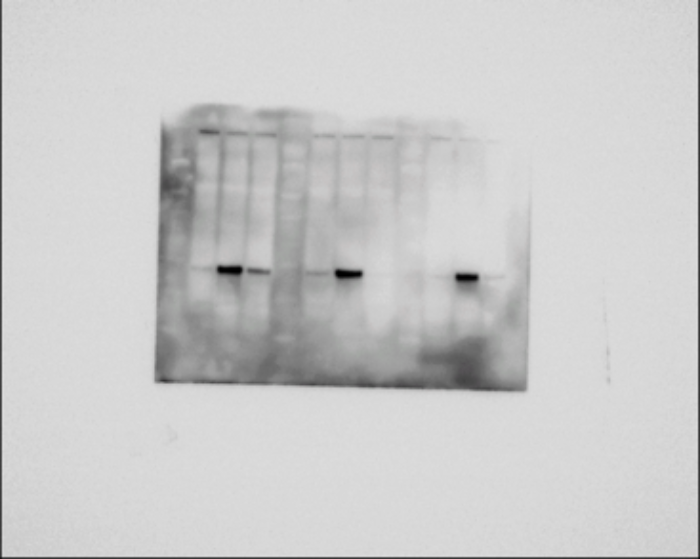

Supplement: Figure 7—source data 3. [file elife-105264-fig7-data3.zip › Fig7c anti-HA.tif]

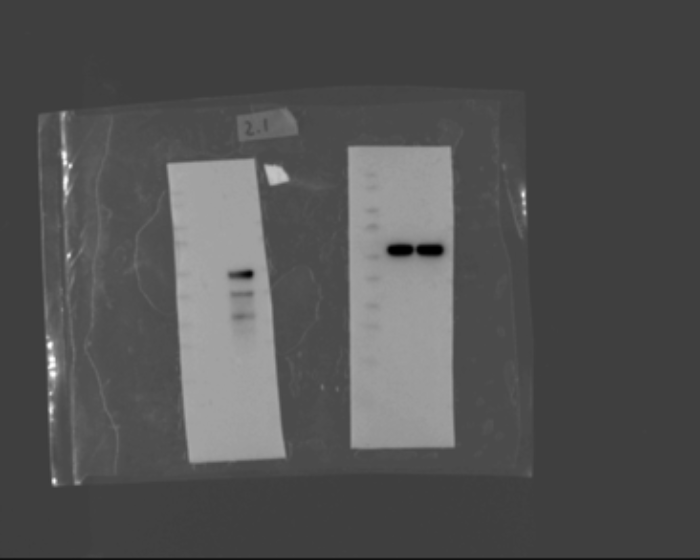

Supplement: Figure 7—source data 3. [file elife-105264-fig7-data3.zip › Fig7c anti-FLAG tubulin.tif]

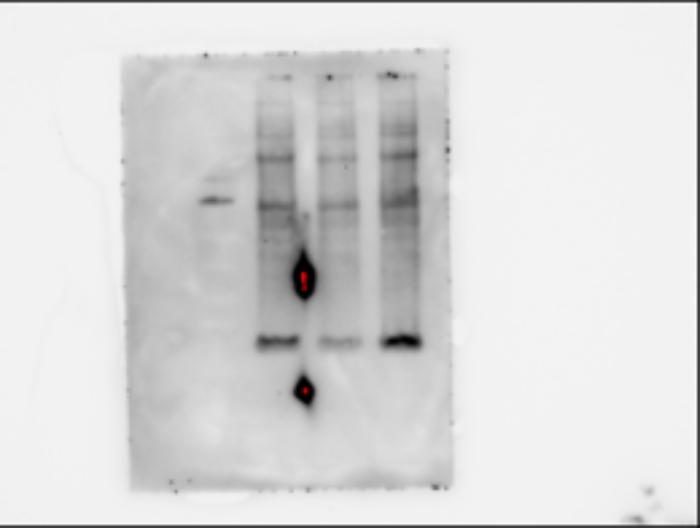

Supplement: Figure 7—source data 3. [file elife-105264-fig7-data3.zip › Figure 7d anti-Arf1.tif]

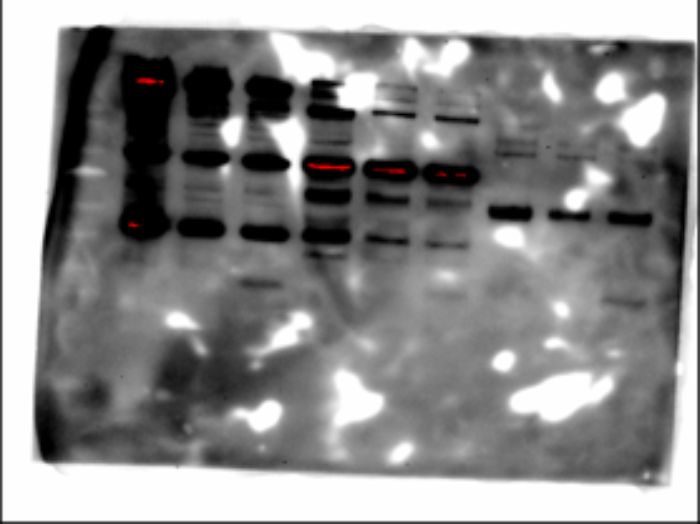

Supplement: Figure 7—source data 3. [file elife-105264-fig7-data3.zip › Figure 7d anti-FLAG.tif]

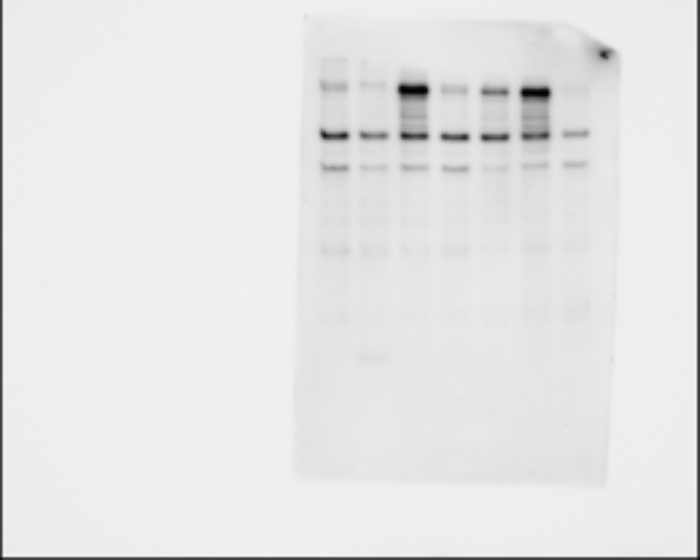

Supplement: Figure 7—source data 3. [file elife-105264-fig7-data3.zip › Figure 7d anti-TfR.tif]

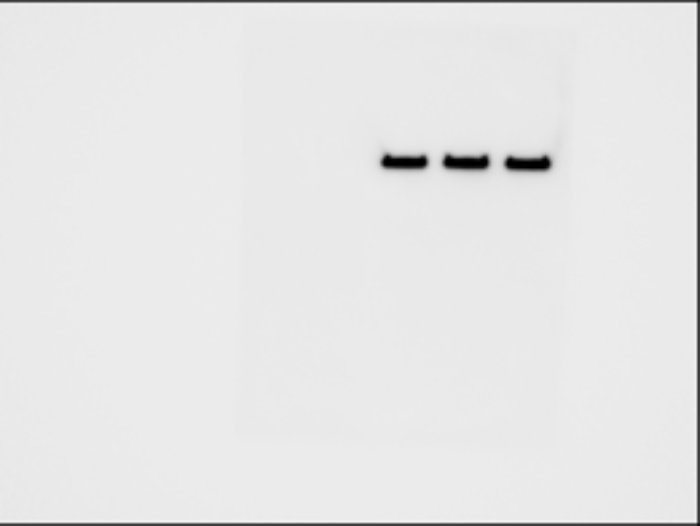

Supplement: Figure 7—source data 3. [file elife-105264-fig7-data3.zip › Figure 7d anti-Tubulin.tif]
