# Supplementary material for: A Commander-independent function of COMMD3 in endosomal trafficking
Source: eLife. 2025 Aug 21;14:RP105264. doi: 10.7554/eLife.105264 (PMC12370252; doi:10.7554/eLife.105264)
Supplement: Figure 7—source data 2. [file elife-105264-fig7-data2.zip › Figure 7-Source data 1.pdf]

Fig. 7c

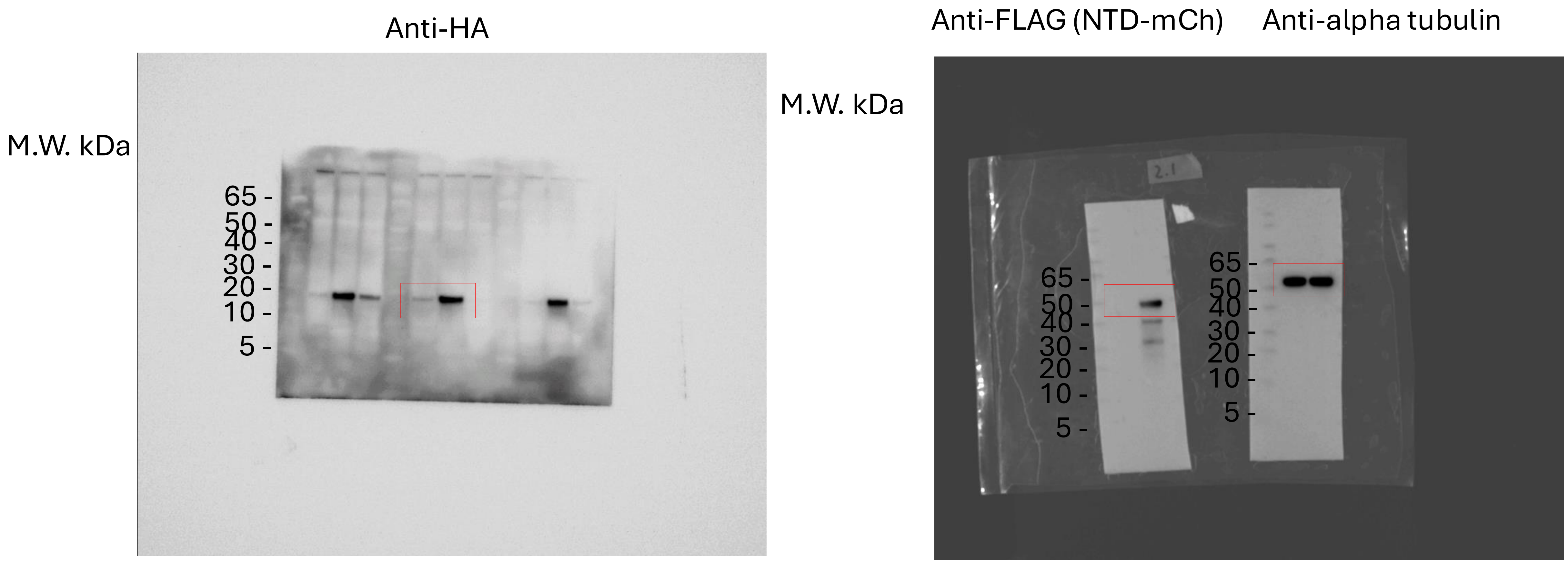
Anti-HA
M.W. kDa
65 -
50 -
40 -
30 -
20 -
10 -
5 -
Anti-FLAG (NTD-mCh)
Anti-alpha tubulin
M.W. kDa
65 -
50 -
40 -
30 -
20 -
10 -
5 -
65 -
50 -
40 -
30 -
20 -
10 -
5 -

Fig. 7d

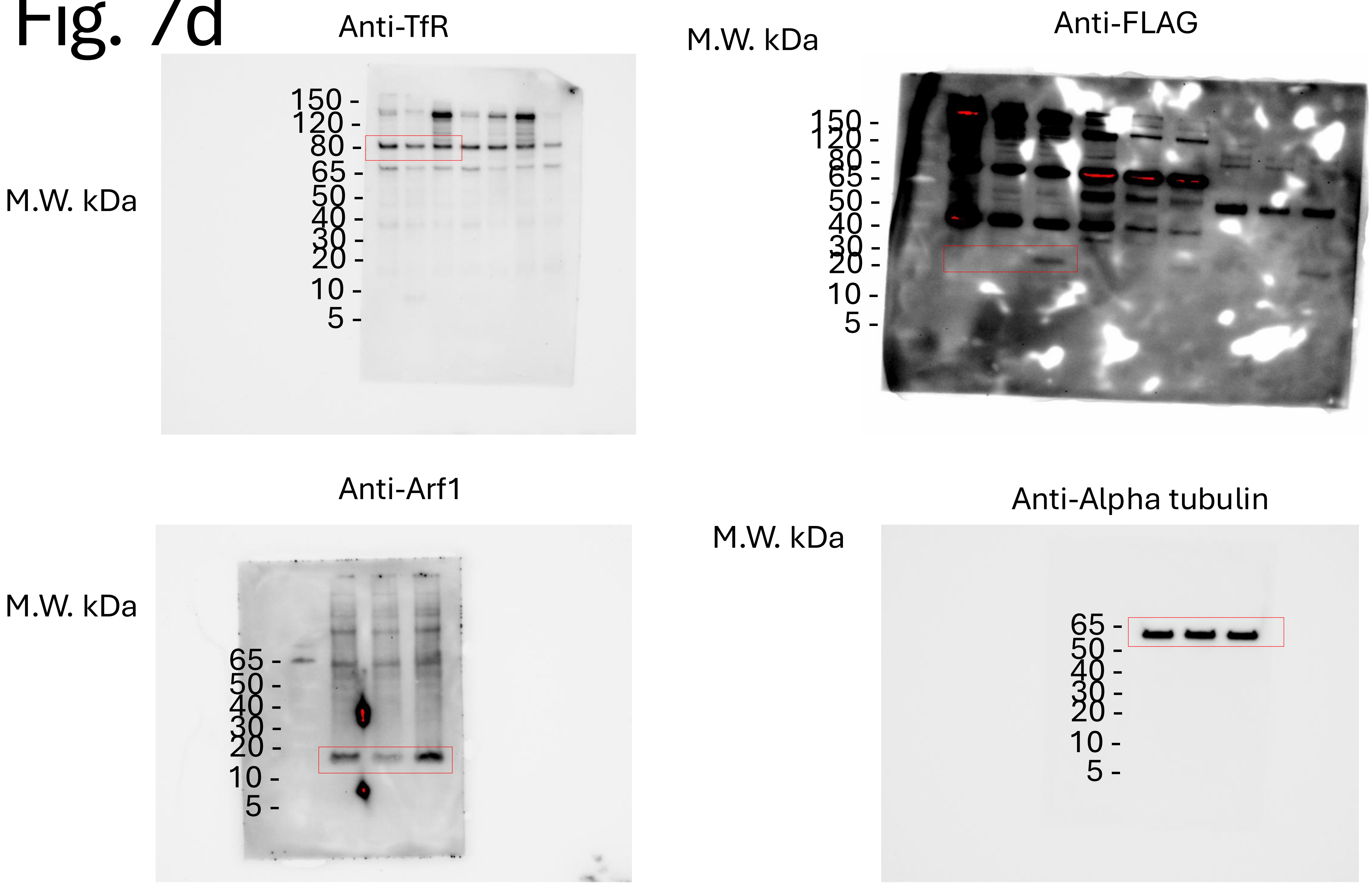
Anti-TfR
M.W. kDa
150 -
120 -
80 -
65 -
50 -
40 -
30 -
20 -
10 -
5 -
Anti-FLAG
M.W. kDa
150 -
120 -
80 -
65 -
50 -
40 -
30 -
20 -
10 -
5 -
Anti-Arf1
M.W. kDa
65 -
50 -
40 -
30 -
20 -
10 -
5 -
Anti-Alpha tubulin
M.W. kDa
65 -
50 -
40 -
30 -
20 -
10 -
5 -
